# Left-right axis asymmetry determining human *Cryptic* gene is transcriptionally repressed by Snail

**DOI:** 10.1186/s12861-016-0141-x

**Published:** 2016-10-28

**Authors:** Kartik Gupta, Vijaya Satish Sekhar Pilli, Gopala Krishna Aradhyam

**Affiliations:** Department of Biotechnology, Bhupat & Jyoti Mehta School of Biosciences, Indian Institute of Technology Madras, Chennai, 600036 India

**Keywords:** Transcription, EGF-CFC, Cryptic, Snail, Left-right-axis

## Abstract

**Background:**

Establishment of the left-right axis is important for positioning organs asymmetrically in the developing vertebrate-embryo. A number of factors like maternally deposited molecules have emerged essential in initiating the specification of the axis; the downstream events, however, are regulated by signal-transduction and gene-expression changes identifying which remains a crucial challenge. The EGF-CFC family member Cryptic, that functions as a co-receptor for some TGF-beta ligands, is developmentally expressed in higher mammals and mutations in the gene cause loss or change in left-right axis asymmetry. Despite the strong phenotype, no transcriptional-regulator of this gene is known till date.

**Results:**

Using promoter-analyses tools, we found strong evidence that the developmentally essential transcription factor Snail binds to the human Cryptic-promoter. We cloned the promoter-region of human Cryptic in a reporter gene and observed decreased Cryptic-promoter activation upon increasing Snail expression. Further, the expression of Cryptic is down-regulated upon exogenous Snail expression, validating the reporter assays and the previously identified role of Snail as a transcriptional repressor. Finally, we demonstrate using gel-shift assay that Snail in nuclear extract of PANC1 cells interacts with the promoter-construct bearing putative Snail binding sites and confirm this finding using chromatin immunoprecipitation assay.

**Conclusions:**

Snail represses the expression of human Cryptic and therefore, might affect the signaling via Nodal that has previously been demonstrated to specify the left-right axis using the EGF-CFC co-receptors.

## Background

Embryogenesis is a process of cooperative and independent stochastic changes being positively driven towards organ formation [1]. Although we understand the process of organ formation in some detail, we lack knowledge of the initial molecular events such as signaling and transcriptional regulation that are triggered towards organ positioning [2]. A central axis in the vertebrate embryo, defining the L-R positioning of organs in the adult is a hallmark of evolutionarily conserved events that give rise to body asymmetry [2, 3]. Organ positioning is orchestrated by genes that are themselves asymmetrically expressed in the early embryo [4]. Differential expression of genes involves initial symmetry breaking events that later choreograph the correct positioning of organs along the L-R axis [4]. Indeed, incorrect positioning of organs along the axis manifests pathologically, often resulting in lethal implications e.g., cardiac abnormalities [5]. Thus studies of molecular mechanisms that cause asymmetric design of the organism’s body are of both, molecular and clinical importance.

Even as the initial set of molecular events leading to asymmetric distribution of body organs is largely unknown, limited understanding of the L-R axis specification includes asymmetric expression of TGF-β family members Nodal and Lefty, along with the transcription factor Pitx2 on the prospective left side of the embryo [6–8]. In contrast, the transcription factor Snail is unique, being the only known transcription factor expressed on the prospective right side of most vertebrate embryos [8]. This highlights the ability of Snail to control gene expression to the prospective right side of the organism and emphasizes the need to identify the very initial events and genes that are regulated by Snail leading to asymmetric positioning of the organs.

As part of the Nodal signalling, the EGF-CFC family [comprising of Cripto, Frl1 and Cryptic (CFC1)], is emerging as an important determinant of the body axes [9, 10]. Human Cryptic encodes a 224 amino acids long protein consisting of an N-terminal signal sequence, C-terminal hydrophobic region and a novel cysteine rich motif called the CFC-motif along with an EGF-like motif [11]. The important role of Cryptic gene in controlling the L-R axis in humans emerged when mutations were demonstrated to be causal to randomly positioned organs in patients, many leading to congenital heart defects including transposition of the great arteries [12–16]. Given that L-R axis defects and defects in laterality occur at a significant frequency (1 in 8500 live births), the genotype-phenotype relationship emanating from the Cryptic expression pattern is an important process to understand [13, 14].

Transcription factor Snail is a crucial repressor of gene expression in early stages of embryogenesis as understood in mice, where it binds to the Snail binding element including CANNTG [16]. Snail acts at the ectoderm-mesoderm boundary repressing genes meant for ectodermal specification and leading to mesoderm formation [17]. It is known to promote epithelial to mesenchymal transitions (EMTs) by controlling a number of genes [18]. Recently, our group identified Cripto-1 as another EGF-CFC member that is repressed by Snail, concomitant with the related EMT gene-changes [19]. Mutations in Snail, interestingly, have been reported to be involved in abnormalities with the L-R axis specifications in mice [7, 8].

Indirect evidences like (i) overlapping temporal expression during early (pre-somite) stages of mouse embryogenesis (ii) similarity in the phenotypes of the respective mutants and (iii) Snail and Crytpic genes control the formation of mesoderm suggest that Snail might be transcriptionally regulating Cryptic levels. Here we demonstrate, both in vitro and in vivo, that the transcription factor Snail binds to the promoter region of Cryptic gene and represses its expression. Our study suggests a putative molecular mechanism by which the initiation of the formation of L-R axis might be established via the transcriptional regulation of Cryptic gene expression by Snail.

## Results

### Identification of Cryptic promoter region and its putative transcription factors

Although there are reports demonstrating specific expression patterns of Cryptic gene in the mouse-embryos and adults, information about its transcriptional regulation and controlled expression is missing [13–15]. In order to identify the transcription factors that might bind to the promoter region of human Cryptic, we utilized computational and bioinformatics tools.

**Fig. 1:**
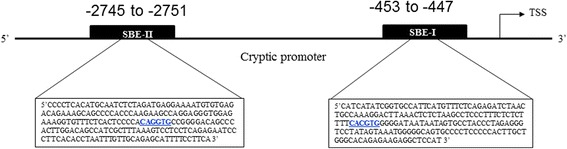
Genomic organization of Cryptic gene and putative Snail binding sites in the promoter region. Nucleotide sequence of Cryptic promoter region is shown in the boxes. The Cryptic promoter sequence was analysed for the Snail binding elements (*SBE*). Schematic location of the two predicted Snail binding sites- CACGTG (at −453 to −447 bp relative to TSS, *SBE1*) and CAGGTG (at −2745 to −2751 relative to the TSS, *SBE2*) is represented. TSS: Transcription Start Site

The human Cryptic gene, 6.8 kb in length, is oriented negatively on the long arm of chromosome 2 (2q21.1) and is composed of 6 exons with predicted splice variants [12]. For this study we retrieved the human Cryptic promoter sequence (from −2.8 kb to +20 bp relative to the transcription start site) from Ensembl (www.ensembl.org) and identified putative transcription factors that might bind on this sequence, using the online database “ConSite” (http://consite.genereg.net/). The search led to the identification of one binding site for Snail (a known transcription factor) while manual screening retrieving another putative site at position −447 bp (Fig. 1), referred to as SBEI (proximal to Transcription start site) and SBEII (more distal to Transcription start site).

## Snail binding to Cryptic promoter causes repression of its expression

**Fig. 2:**
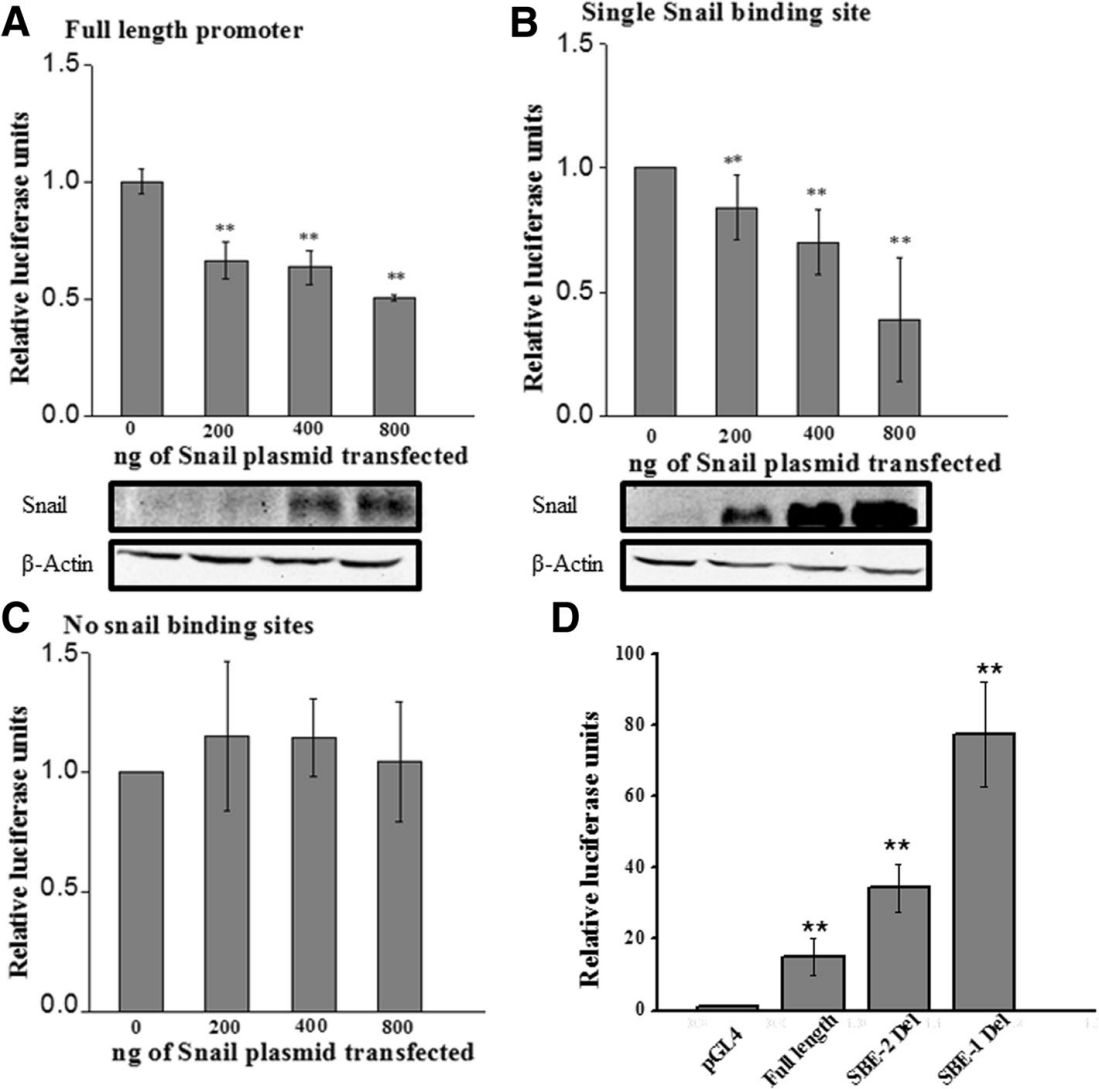
Cryptic promoter activity in cells over expressing Snail. Plasmid construct expressing Snail was transfected in increasing concentrations (as indicated) in HEK 293 cells along with reporter constructs for Cryptic promoter activity by cloning the Cryptic promoter region upstream of the firefly luciferase gene. (**a**) The full length Cryptic promoter, (**b**) Promoter region containing a single Snail binding element (*SBE1*), and (**c**) deleted Snail binding elements are co-expressed with increasing concentrations of the vector expressing Snail. (**d**) Luciferase acivity is also measured for the Cryptic promoter construct either containing SBEI or SBEII mutant or full-length and for the vector alone. Empty reporter vector is used as vector control, pCDNA3 is used as control for Snail transfection and Beta-galactosidase construct is utilized to ensure equal transfection. The relative luciferase activity is plotted as a function of increasing Snail expression. Experiments are carried out in triplicates and repeated at least 3 times. Data with *p* < 0.05 is considered significant

To study the influence of Snail in regulating the expression of the Cryptic gene (activation or repression) we assayed for the interactions between Snail protein and the Cryptic gene promoter in a exogenous system. We sub-cloned the human Cryptic promoter including the transcription start site upstream of a luciferase gene in the PGL4.20 vector. Relative luciferase activity was measured in HEK-293 cells by co-transfecting Snail expression plasmid and the promoter containing vector in different ratios. We performed these gene reporter assays as described earlier [20]. The colorimetric measurements (i.e., the luciferase read-out) are directly related to the strength of the promoter and help in identifying the promoter activity influenced by the interactions. Luciferase activity of Cryptic promoter is attenuated by the co-transfected Snail in a dose dependent manner (Fig. 2a, b and c). This reduction in luciferase activity demonstrates that Snail represses the promoter activity (correlated to gene-expression) of the Cryptic gene. Further, successive deletions of the two putative Snail binding sites i.e., SBEI and SBEII (Fig. 2 b, c and d) from the promoter site led to a step wise recovery of Cryptic promoter mediated luciferase expression again demonstrating that Snail influences the promoter activity of Cryptic by directly interacting with its promoter.

## Cryptic expression is negatively regulated by Snail

**Fig. 3:**
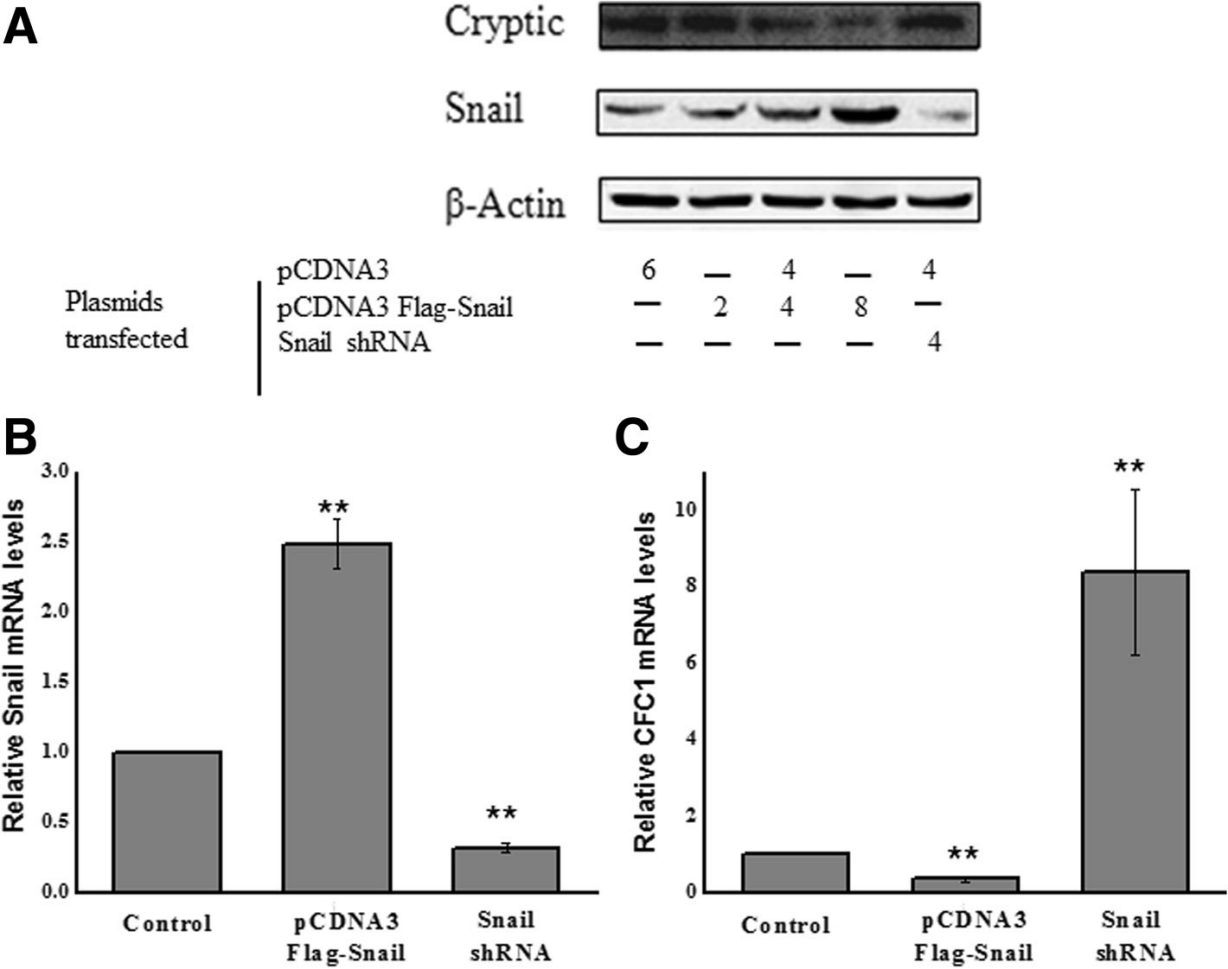
Endogenous Cryptic levels are attenuated by Snail expression and are restored upon Snail depletion in PANC1 cells. **a** Cryptic and Snail levels are measured by western blotting after transfecting different amounts of Snail/control/shRNA plasmids (2/4/6 μg of Snail plasmid and 4 μg of shRNA plasmid and total amount of plasmid made up to 8 μg with pCDNA3 empty vector). Equal loading is confirmed by beta-actin. The blot is representative of 3 experiments (*n* = 3). qPCR is performed on reverse transcribed samples to estimate the mRNA levels of (**b**) Snail and (**c**) Cryptic and is normalized to beta-actin expression (*n* = 4)

We next wanted to measure the levels of Cryptic in response to Snail expression to directly test whether the reduced promoter-activation observed in the luciferase assays also corresponds to the de-novo Cryptic-protein levels. An existing challenge remains on obtaining sufficient endogenous levels of Cryptic in non-embryonic cells. We utilized a differentiation system reported before (Patent CA2719385A1); briefly, PANC1 cells were differentiated in-vitro and as expected, Cryptic levels were found up-regulated (data not shown). Using this, we exogenously expressed Snail at various ratios and assayed for endogenous Cryptic-expression by western blotting (Fig. 3a). We also used a loss-of-function approach by using an shRNA targetting Snail to test the effect on Cryptic-expression. Consistent with our hypothesis, we found that exogenous expression of Snail repressed Cryptic protein measured by western blotting. Upon 2 μg, 4 μg and 6 μg of pCDNA3 human Snail plasmid transfection Cryptic expression was reduced by 30 % ± 10 %, 56 % ± 11.8 % and 80 % ± 8.9 %. (Fig. 3a). Further, silencing Snail resulted in restored expression of Cryptic. 4 μg Snail shRNA reduced the Snail expression by 70 % ±10 % and enhanced Cryptic expression by 59 % ± 12 %,validating that Snail negatively regulates Cryptic-expression (Fig. 3a).

We then used qPCR analyses using over-expression and shRNA mediated knockdown of Snail and tested its effect on Cryptic mRNA expression. We verified that the Snail over-expression and knockdown indeed led to change in Snail transcripts (Fig. 3 b) and its effect on Cryptic mRNA levels (Fig. 3c). We observed that overexpression of Snail led to a decreased mRNA of Cryptic whereas knocking down Snail led to increased Cryptic mRNA (Fig. 3c).

We conclude from the western blotting and qPCR assays that the abundance of Snail causes attenuation of Cryptic expression that can recovered by depleting Snail.

## Snail interacts with Cryptic promoter in vitro

**Fig. 4:**
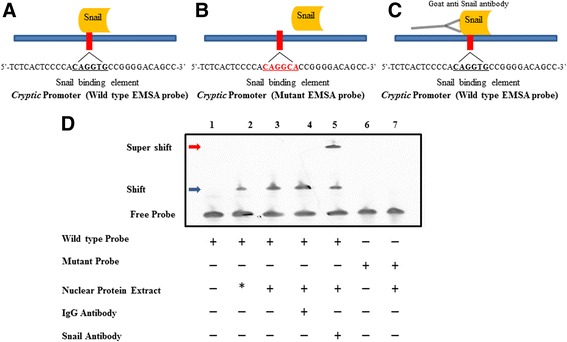
Interaction of Cryptic promoter region with endogenous Snail in nuclear lysates of PANC1 cells. Total nuclear protein extract (NPE) was isolated from PANC1 cells that express Snail endogenously. (**a**) Schematic of oligonucleotide duplex corresponding to Cryptic promoter region 15 bp upstream and 15 bp downstream of the Snail binding element was used for electrophoteric shift, (**b**) Schematic of the Snail binding element was mutated (showed in red). (**c**) the schematic complex of oligonucleotide supershift is depicted. (**d**) *Lane 1* represents the biotinylated probe. *Lanes 2* and *3* represent the incubation of increasing amounts of NPE with wild type probe. *Lanes 4* and *5* are obtained upon incubating the NPE with the wild-type oligonucleotides with IgG control or Snail specific antibodies. *Lanes 6* represents the mutated Snail binding element *Lane 7* represents the incubation of the NPE with SBE mutated oligonucleotide. NPE: nuclear protein extract, * represents 10 μg NPE; blue and red arrows represent shift and supershifts, respectively

To ascertain whether the repression of Cryptic is based on the binding of Snail with the Cryptic promoter, we assayed the in vitro interaction between the two by Electrophoretic Mobility Shift Assay (EMSA). Total nuclear protein extract (NPE, containing endogenous Snail protein) was obtained from PANC1 cell line. Oligonucleotides (wild type and mutated sequences of 30 bp in length), corresponding to the sequence of the Cryptic promoter and the putative Snail binding site were used for studying these interactions (Fig. 4). Interaction between NPE and the wild-type oligonucleotides led to retardation in the movement of oligonucleotides on the gel that was dependent upon the amount of NPE (Fig. 4, Lane 2,3) indicating the formation of a high molecular weight complex and therefore interaction between them.

To confirm that the binding factor in the NPE is indeed Snail, the specificity of interaction was ascertained by incubating NPE and oligonucleotide complex with Snail antibody (~3 μg) or with IgG control antibody (~3 μg) (Fig. 4, Lanes 4,5). Relative to the bands obtained upon incubation of NPE with the oligonucleotides we were able to observe a supershift in the band intensity only with Snail-antibody whereas IgG control did not cause such a shift (Fig. 4, Lane 4,5). The supershift indicates the formation of a ternary complex between the oligonucleotide, the Snail protein and the antibody. We confirm the same by using another Snail-specific antibody that demonstrates the presence of a faded band (data not shown), owing to the competition between the oligonucleotides and the antibody for Snail protein. Further, the specificity of the interaction was confirmed by a loss in interaction when the NPE is incubated with mutant oligonucleotides (Fig. 4, Lane 5, 6), suggesting that a factor from the NPE interacts with the Cryptic promoter at the Snail binding site. We therefore conclude that Snail specifically interacts with the Cryptic promoter even when the interaction is reconstituted in vitro.

## In vivo interaction between Snail and cryptic promoter

**Fig. 5:**
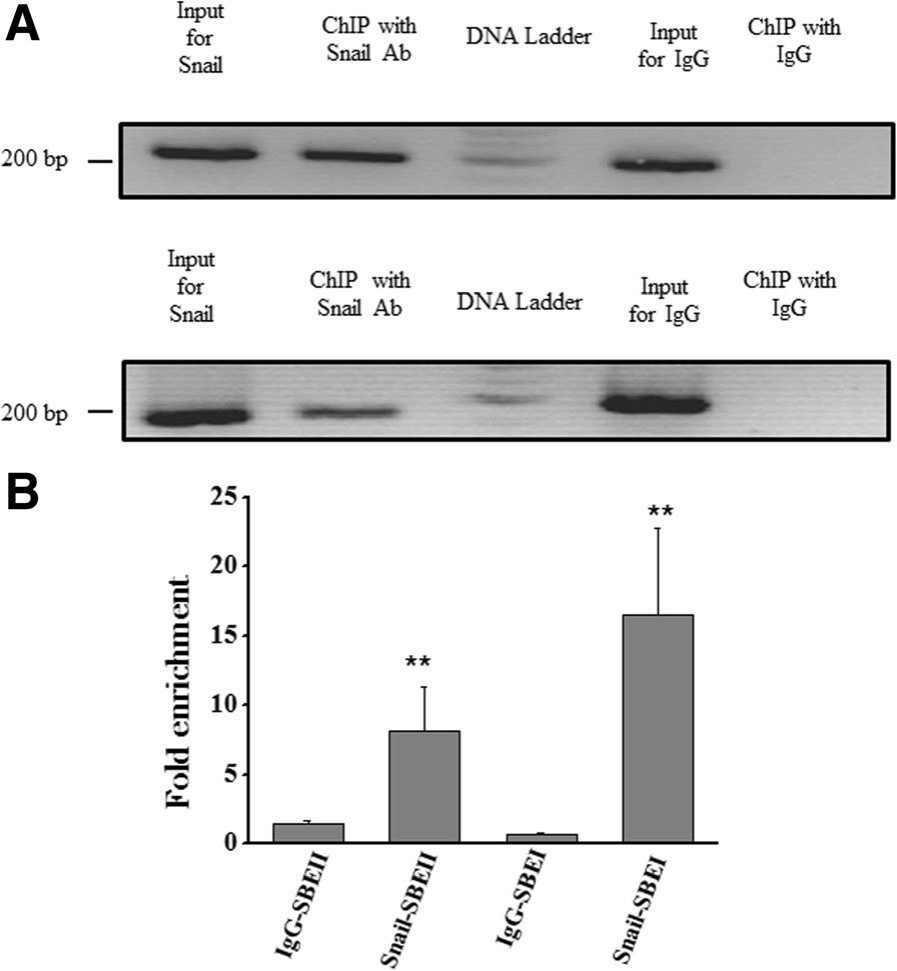
Interaction of Snail with the Cryptic-promoter in-vivo. Chromatin Immunoprecipitation (ChIP) was performed in PANC1 cells for the two putative Snail binding sites using **a**) semi-quantitative or **b**) qPCR. The cells expressing endogenous Snail were cross linked using formaldehyde followed by shearing and immunoprecipitation using a Snail specific or IgG control antibody. The resulting chromatin was reverse cross linked and amplified using the primers flanking the two putative Snail binding sites. Equal loading was confirmed by the amplification of input chromatin. The resulting blot (4A) and the quantification (4B) is representative of 3 experiments (*n* = 3)

Interaction of Snail and Cryptic promoter was also assayed in vivo using chromatin immunoprecipitation (ChIP). Briefly, cross-linking of total-protein and DNA was performed using formaldehyde in PANC1 cells that express Snail endogenously. The DNA obtained in the chromatin immunoprecipitate using Snail specific or control (IgG) antibody was assayed utilizing respective primer sets for the two binding sites of Snail on the Cryptic promoter by both semi-quantitative PCR and qPCR. PCR analyses of these products revealed an amplification of the samples corresponding to the Snail specific antibody for both the Snail binding sites along the Cryptic promoter (Fig. 5a &b). In contrast, no amplification for the nonspecific control (IgG antibody) was observedthereby (Fig. 5 a & b) confirming that Snail indeed binds to the Cryptic promoter in vivo.

## Discussion

The emergence of complex embryonic pattern and cellular differentiation eventually leading to the development of body formation are of utmost beauty, enigma and importance in biology. Understanding the molecular basis of initiation and establishment of the L-R axis asymmetry in an embryo remains a great challenge in the field of developmental biology [3, 21, 22]. Studies regarding asymmetric regulation of a number of signalling proteins, motor proteins and transcription factors involved in establishing this asymmetry have aided our understanding to some extent. Among the genes studied, the importance of the highly conserved EGF-CFC family of genes is yet to be realised [10, 23]. Probably through gene duplication and specification, the mammalian genome contains two of the EGF-CFC family members Cryptic and Cripto, each controlling different cellular functions [10, 23]. Mutations detected have helped to conclude that ‘Cripto’ controls the anterior-posterior axis formation in mice, whereas the other member Cryptic, controls the L-R positioning of organs in humans indicating a divergence in the function of the two family members [10].

Studies on Cripto have focused majorly on its aberrant expression during cancer with preliminary understanding of its transcriptional control [24]. Expression of Cryptic protein, on the other hand, is observed transiently during early embryogenesis whereas, in adults, it is confined to a few organs in mice and humans [11]. The indispensable role played by members of this family in specifying the axes during embryonic development and the functional diversity between these genes warrants the study of upstream regulators of their expression. To this end, some details are known about Cripto, but so far no transcriptional regulators have been reported for Cryptic.

Snail is a zinc-finger and basic helix-loop-helix containing transcription factor that represses genes involved in the formation of the ectoderm and is indispensable for the formation of mesoderm [7]. It is involved in the movement of cells in the developing embryo and its aberrant activation leads to several metastatic cancers [25]. As a transcription factor, Snail represses the expression of epithelial markers like E-cadherin, thereby conferring motility to cells. Since it is expressed non-uniformly [4] across the L-R axis, Snail controls the spatial expression of genes (for example Pitx2) rendering positional information to cells in the developing embryo [4]. It is likely that Snail not only controls the expression of genes involved in motility directly, but also controls genes that convey positional information to the developing organs like heart.

In mouse and humans, Cryptic gene has been demonstrated to be responsible for the establishment of the L-R axis [10, 14]. Defects/mutations in the Cryptic gene cause abnormal positioning of visceral organs, leading to congenital heart defects in humans and inversion of major blood vessels, among other clinical phenotypes [10, 14–16]. Cryptic has also been implicated in the formation of the mesoderm, which is the layer that is formed through delamination and mass movement of cells from the ectoderm [11]. Despite our knowledge that the CFC-family is involved in control of axis formation in the developing embryo, the regulation of gene expression for Cryptic (a typical member) is completely unknown. The absence of knowledge of the upstream regulators of Cryptic impedes our understanding of its role in a broader scale of developmental events.

To identify the transcription factors that control human Cryptic expression, we computationally predicted that Snail binds to its promoter region and validated the significance of changes to promoter activity using luciferase assays (Fig. 2). Here we demonstrate that Snail expression suppresses the Cryptic gene when they are co-transfected into HEK-293 cells in a dose dependent manner. This also correlates with decreased Cryptic expression upon exogenous Snail expression (Fig. 3). We also demonstrate in vitro (through EMSA experiments) interaction of Snail with promoter region of the human Cryptic gene (Fig. 4). The specificity of this interaction is revealed by competition assay and super shift experiments. We confirm our observations by demonstrating in vivo binding of Snail to Cryptic promoter using ChIP assay (Fig. 5). These results establish that Snail, as a transcription factor, negatively regulates Cryptic gene by direct transcriptional repression.

Our finding that Snail acts upstream in the Cryptic mediated signalling is an important advancement in understanding the basis of L-R axis specification. Snail is unique in its expression on the prospective right side of the developing mouse embryo and conditional knockouts cause phenotypes that are surprisingly similar to Cryptic mutants, including reversed positioning of the outflow tract of heart and reversed looping of the heart [10]. We now provide Snail mediated Cryptic repression as a mechanism likely to account for the observed similarity in phenotypes.

**Fig. 6:**
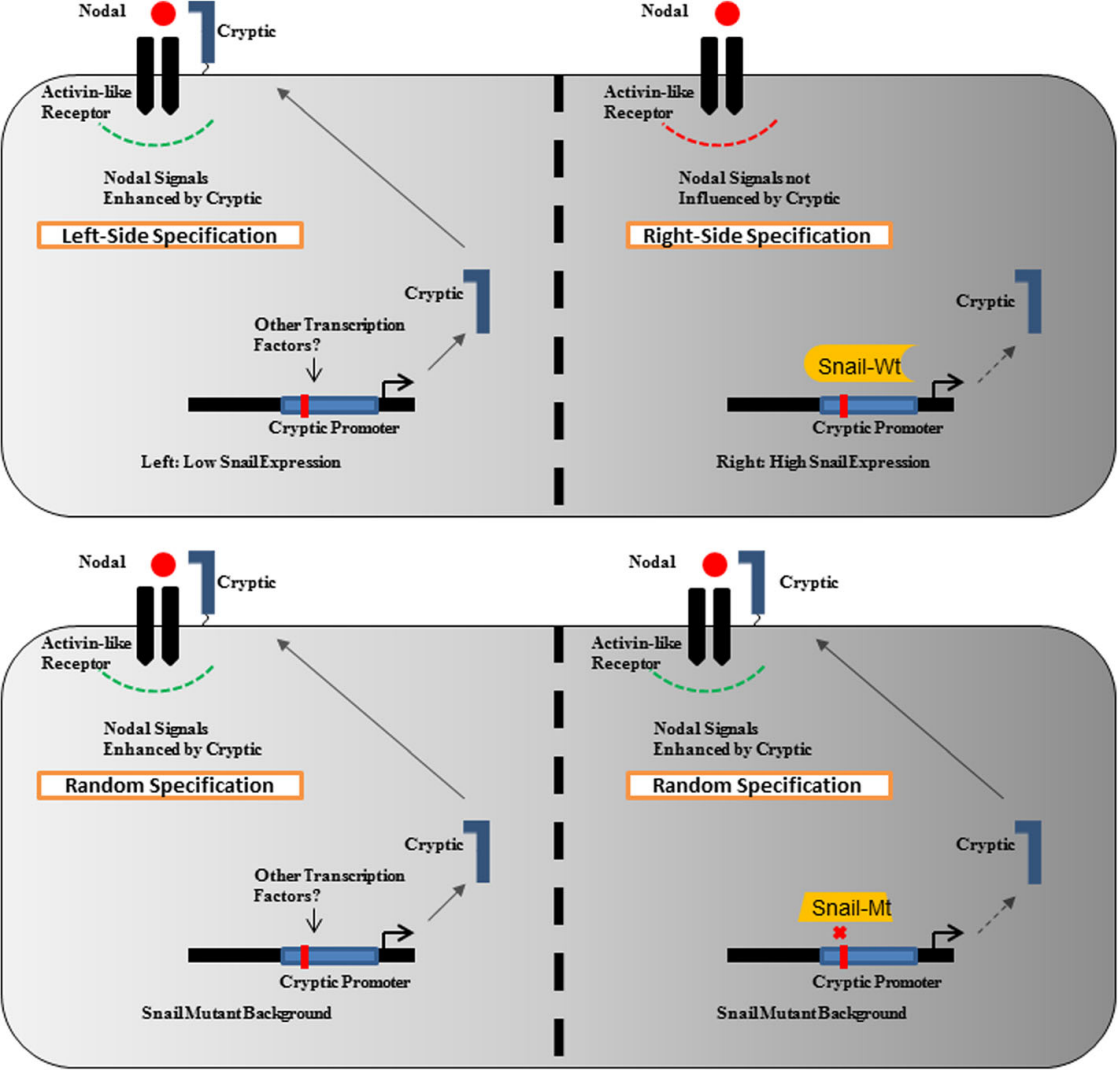
Proposed mechanism of Snail mediated L-R axis specification through Cryptic repression. *(Up,left)* Low endogenous expression of Snail on the left side of the developing embryo permits Cryptic-mediated Nodal signalling, causing left-side specification. (*Up, right*) Relatively higher levels of Snail on the right side suppress Cryptic-mediated Nodal signalling resulting right-side specification. *(Bottom)* A Snail mutant background is reported to aberrantly activate Nodal signalling. The de-repression of Cryptic in a mutant Snail background may cause bi-laterally symmetrical activation of Nodal signalling and thereby random organ positioning

Further, the significance of Snail mediated repression of Cryptic is evident by the spatial patterns of Nodal expression in Snail mutant embryos [21, 26]. While Nodal is normally expressed on the left side of the embryo, Snail mutant embryos display a bilateral expression of Nodal. This may be explained by the fact that Snail negatively regulates Cryptic expression. Nodal signalling, being promoted by Cryptic, demonstrates that it acts as a co-factor for Nodal [21, 26]. It might thus be expected that absence of Cryptic causes decreased Nodal signalling. Thus, in Snail mutant mouse embryos, the aberrant activation of Nodal might be a result of the de-repression of the control exercised by Snail over Cryptic, thus promoting Nodal signalling (Fig. 6).

Experiments on chick embryos have illustrated that the Snail expression is dominant in controlling the formation of the pro-epicardium by repressing Pitx2, similar to our observation of Cryptic repression [21, 26]. The development of normal, right-sided pro-epicardium in chick embryos was observed to remain unaffected upon manipulating Nodal or Cryptic, but the artificial (ectopic) expression of Snail (where it is normally not-expressed) caused the abnormal formation of the pro-epicardium at that site. Thus, Snail is likely to repress Cryptic expression in a way similar to the repression of Pitx2 [21, 26].

Additionally, Nodal expression in chick embryos is not affected by high levels of Snail anti-sense oligonucleotides [8]. Our finding that Cryptic, a co-receptor for Nodal signals, is repressed by Snail now provides a plausible mechanism behind this observation. In the absence of Snail, freely transcribed Cryptic causes propagation of Nodal signals by acting as its co-receptor. With this mechanism in action, addition of high levels of Snail anti-sense oligonucleotides will have no effect on Nodal (as shown in this report) because Cryptic repression is already withdrawn. On the contrary, it is likely that *high* expression of Snail suppresses Nodal signalling.

In future, it may be interesting to find out the relevance of the interaction of Snail with Cryptic gene at a cellular level. The participation of Snail and Cryptic in the TGF-beta pathway might prove to be a point wherein Snail and Cryptic interaction has this physiological role. With further identification of the transcriptional regulators of Cryptic, we are likely to be able to associate more clinical cases of incorrect organ positioning with defects in the regulators of the Cryptic gene.

## Conclusion

Snail and Cryptic are essential for left right asymmetry in mammals. In present study we demonstrated that over expression of Snail suppresses Cryptic expression in transdifferentiated PANC1 cells. Through promoter binding studies and luciferase assays we confirmed that Snail directly binds to Cryptic gene promoter and regulates its expression. Our study has implications in the establishment of the left-right axis asymmetry where the gene-regulatory mechanism described in this report may be utilized.

## Methods

### Plasmids and antibodies

Fragment spanning −2800 to +20, relative to the transcription start site of human Cryptic gene sequence (accession number: NC_000002.11) was PCR amplified with primers (Forward: 5′GGTACCCCCCTCACATGCAATCTCTAG3′ and Reverse: 5′CTCGAGCTCTATGAGACCT GGCTGGG3′ flanking *Kpn*I and *Xho*I sites) (GenoRime, India) with reaction conditions set as: 98 °C for 5 min, 98 °C for 30 s, 64 °C for 30 s, 72 °C for 3 min, repeat cycle 2–4 × 30 times, 72 °C for 15 min, hold at 4 °C and the amplified product cloned into pGEMT Vector (Promega, USA) by TA-cloning to produce pGEMT-CrypticPro. pGEMT-CrypticPro was subsequently sub-cloned into pGL4.20 vector (Promega, USA) using *Kpn*I and *Xho*I sites, to generate pGL4.20-Cryptic luciferase reporter. Further, deletion constructs were made in the similar way by changing the forward primer. Forward primer for single binding construct: 5′-GGTACCCCTCTTGATGGCAAACAGG-3′, for no snail biding site: 5′-GGTACCCGTGCTTTCCCTTATCCTCG-3′. pCDNA3-Flag-Snail is kind a gift from Dr. Weiss (University of Michigan, USA). β-gal plasmid is kind gift from Dr Mahapatra IITM. Mouse monoclonal anti human Cryptic antibody was purchased from R&D systems (MAB1410-SP) and Goat polyclonal anti Snail antibody was purchased from SantaCruz biotech (sc-10433 X).

## Cell culture

HEK-293 and PANC1 cells were procured from the National Centre for Cell Sciences (Pune, India) and were grown in DMEM-high glucose (Gibco, USA), supplemented with 10 % fetal bovine serum (Gibco, USA) and 1 % antibiotics (Anti-Anti, Gibco, USA) in a humidified chamber with 5 % CO_2_.

### Luciferase reporter assays

Promoter strength was tested based on the principle that the luciferase enzyme production that is quantified colorimetrically is dependent on the activity of the promoter and therefore is indicative of the gene driven by promoter. Luciferase assay was performed in 12 well plates after 24 h of co-transfection with pGL4.20-Crypticpro and transcription factor construct (pCDNA3 Flag-Snail) into HEK-293 cells. As control, pCDNA3 plain vector was transfected to ensure that equal amounts of total DNA was transfected in cells. the transfection efficiency was normalized by co-transfection followed by colorimetric measurement of β-Galactosidase activity using ortho-Nitrophenyl-β-galactoside (ONPG) substrate at 420 nm after incubation for 30 min at 37 °C. The luciferase activity was recorded in a fluorescence reader with absorbance maxima at 560 nm. All experiments were repeated at least 3 times, and performed in triplicates. Mean Values +/− standard deviations are reported.

## Western blotting and qPCR

Cells were lysed in radioimmunoprecipitation (RIPA) buffer and protein estimated by BCA kit (Thermo scientific, USA). 100 μg of total lysate loaded on 12 % polyacrylamide gel, transferred on nitro-cellulose membrane (Membrane Solution, USA), blocked with 5 % milk in Tris-buffered saline with Tween-20 (TBST) for 45 min, washed with TBST (3 washes, 5 min each) and were incubated overnight in primary antibodies at 1:5000 dilution: Snail and Cryptic. Next day, blots were washed with TBST (3 washes, 5 min each) and were incubated in HRP-conjugated secondary antibodies. After washing (3 washes, 5 min each), blots were developed in Versa Doc (Biorad, USA). Equal loading was confirmed by normalizing with β-actin (Santa cruz Biotechnology, Inc, USA).

To evaluate the expression of Cryptic and Snail, primers were purchased from Qiagen (catalog number— QT00070287) and for Snail as described in Pilli et al. (2015) (19). Briefly, total RNA was converted to cDNA using reverse transcriptase as described before (19). qPCR as performed as per manufacturer’s instructions using SYBR Green polymerase master mix (Applied Biosystems). Results were analyzed using ΔΔCt method (see Applied Biosystems—qPCR support page).

## Chromatin immunoprecipitation assays

Chromatin immunoprecipitation was performed as published earlier [27]. Briefly, transcription factors and the DNA were cross linked by incubating the cells in fixing solution (1.35 % formaldehyde) for 12 mins. Fixing reaction was terminated by adding 0.12 M Glycine in PBS. After fixing, cells were scraped in scraping solution (PBS + 0.01 M EDTA+ 0.1 μM PMSF) and lysed in lysis buffer (50 mM Tris pH 8.1, 1 % Triton ×100, 0.1 % Sodium-Deoxycholate, 5 mM EDTA, 150 mM NaCl, 1 % SDS) and nuclear fraction collected by spinning the sample at 3000 rpm for 7 mins. Nuclear fraction was sonicated to shear the chromatin. Approximately 10 μg of chromatin was incubated with 2 μg antibody against Snail for 12 h after pre clearing the chromatin with 30 μl of protein A/G beads (Santacruz). Chromatin – antibody complex was pulled down with protein A/G beads after 4 h incubation of 60 μl protein A/G beads with chromatin. DNA:protein immune complexes were eluted, cross linking reversed and DNA was extracted. The presence of Cryptic promoter domains containing binding elements in immunoprecipitated DNA was identified by PCR using the following primers: SBE-II: forward, 5′-AAAGGGCCAGGTAGAAAACAT-3′; reverse, 5′GTTTGGTAATGCCCAAAAGCT-3′; SBE-I forward: 5′-CATCATATCGGTGCCATTCA-3′ and SBE-I reverse: 5′-ATGGAGCCTCTTCTCTGTGC-3′. The PCR conditions were as follows: 98 °C-2 min, 98 °C −30 s, 58.6 °C −30 s, 72 °C −30 s, repeat step 2–4 (30 cycles), 72 °C-3 min and final hold at 4 °C). Quantitative PCR was performed using SYBR Green polymerase master mix (Applied Biosystems).

## Electrophoretic Mobility Shift Assays (EMSA)

Oligonucleotides of 30 bp (flanking +/− 15 bp of consensus site and mutant consensus sequence) were synthesized (GenoRime, India). Wild type forward sequence is 5′-TCTCACTCCCCACAGGTGC CGGGGACAGCC-3′, mutant forward: 5′- TCTCACTCCCCACAGGCATCGGGGACAGCC -3′ Oligonucleotides were labelled at 5′ end with biotin dUTP with the help of terminal transferase (NEB- M0315S), and annealed used for supershift assays. Control (Goat IgG) antibody was used at levels matching amount of protein used.

### Preparation of nuclear protein extract

Nuclear Protein Extracts (NPE) were prepared using PANC1 cells and estimated for protein concentration using Lowry’s Method. Briefly, cells were grown to confluency under appropriate conditions and harvested after PBS wash, and lysed in PBS by scraping. Cells were pelleted by centrifugation at 6000 rpm for 5 min followed by resuspension in cytoplasmic extraction buffer (50 mM Tris Ph-8.1, 1 % Triton × 100, 0.1 % Na-deoxycholate, 5 mM EDTA, 150 mM NaCl, 1 % SDS and protease and phosphatase inhibitors) and left on ice for 30 min with intermediate vortexing. After incubation, the cytoplasmic content was centrifuged at 14,000 rpm and nuclear content collected as a pellet which was then washed thrice with cytoplasmic extraction buffer. Nuclear content was incubated with nuclear extraction buffer (50 mM Tris pH 8.0, 10 mM EDTA, 0.01 % SDS, protease and phosphatase inhibitors) on ice for 2 h by intermediate vortexing every 5 mins and centrifuged at 14,000 rpm for 10 mins and the supernatant collected as NPE.

## Preparation of ‘reaction mix’ and run on acrylamide gel

EMSA reaction mix is prepared in 2× binding buffer [1 M HEPES (pH 7.9, 360 ul, 24 mM) 1 M TrisHCl pH 8.0, 120 ul, 8 mM), 0.5 M EDTA (pH 8.0, 60 ul, 2 mM), 100 mM DTT (150 ul, 1 mM), dH2O 14.3 ml] with 1 μg/reaction of Poly dI-dC to prevent non-specific interaction. Amount of NPE and Cryptic promoter mimic oligonucleotides used are 0.5 μg of Cryptic promoter mimic oligonucleotides and 20 μg of NPE per reaction. For observing supershift, anti-bodies with weight/weight equivalence to the NPE were used.

Previous day, poly-acrylamide gels were casted for EMSA using 40 % acrylamide (3 ml), 2 % bisacrylamide (2 ml), 10 × TBE (1 ml), TEMED (20 μL), APS (100 μl) and made to 20 ml, and casted on gel-casting apparatus (Biorad). After polymerization, gels were stored at room temperature for 1 h followed by overnight incubation at 4 °C and used next day to ensure complete polymerization. For preparation of reaction mix, 2× binding buffer, poly dI-dC were prepared as a master mix and aliquots stored. NPE was added to the tubes depending upon the reaction, and incubated for 10 min to prevent non-specific binding through poly dI-dC. Afterwards, unlabelled oligonucleotides or anti-bodies were added to the reaction mix. Reactions were prepared to maintain 1 h incubation with unlabelled oligonucleotides and 3 h with anti-bodies. Subsequently, labelled oligonucleotides were added and incubated for 20 min at RT. Loading dye supplied by the manufacturer or Bromophenol blue-Ficol is used for loading. Meanwhile, the poly acrylamide gels are pre-run in 0.5× TBE buffer at 100 V for 30–60 min. The reaction mix was loaded onto the gel and run at 100 V for 50 min or for dye-front to reach the bottom of the gel. Then the gel was stained with Ethidium bromide and scanned for oligo migration.

## Abbreviations

BCA: Bicinchoninic acid assay
ChIP: Chromatin immunoprecipitation
EDTA: Ethylenediaminetetraacetic acid
EGF: Epidermal growth factor
EMSA: Electrophoretic mobility shift assay
EMT: Epithelial to mesenchymal transforming
HRP: Horseradish peroxidase
L-R: Left right
NPE: Nuclear protein extract
ONPG: Ortho-nitrophenyl-β-galactoside
PBS: Phosphate buffer saline
qPCR: (quantitative) Polymerase chain reaction
RIPA: Radio immunoprecipitation assay
RT: Room temperature
SBE: Snail binding element
SDS-PAGE: Sodium dodecyl sulphate- polyacrylamide gel electrophoresis
TBE: Tris/borate/EDTA
TBST: Tris-buffered saline tween 20
TFG-β: Transforming growth factor beta
TSS: Transcription start site
